# Thoracoscopy: The past, the present and the future! A personal journey

**DOI:** 10.7196/AJTCCM.2018.v24i1.182

**Published:** 2018-04-03

**Authors:** I Schewitz

**Affiliations:** Department of Cardiothoracic Surgery, Faculty of Health Sciences, University of Pretoria, South Africa

**Keywords:** thoracoscopy, video assisted thoracic surgery, nuss procedure

## Abstract

Thoracoscopy, in my opinion, is underutilised in Africa, for a multiplicity of reasons. These include a lack of expertise, the perceived cost and difficulties in obtaining and maintaining equipment. The benefits, however, in improved surgery and decreased surgical pain and rapid return to productive work outweigh by far the so-called disadvantages. In my opinion, thorascopic techniques should be routine in all our academic departments. Our newly qualified thoracic surgeons should be trained in video-assisted thoracic surgery.

## Background

Thoracoscopy is over 100 years old. Jacobaeus^[1–3]^ described its use in 1906. He used it mainly for diagnosing tuberculosis pleurisy. With the rise of chemotherapy for tuberculosis, however, it gradually fell into disuse. During the 1970s, gynaecologists started using laparoscopy, as did general surgeons in the late 1980s. The development of fibreoptics, light transmission and image processing has not only made video-assisted surgery more efficient, but has also increased the scope of what is possible. This article is a review of the last 25 years in one South African (SA) practice, and a prediction for the future.

In 1991, Schutte, in the small hospital of Sunward Park, Boksburg, SA, introduced laparoscopic cholecystectomy to the country. He was one of the first in the country to practise the new techniques, which he had learnt in his studies overseas. The advantages were soon obvious in the postoperative course of the patients, who were discharged in 24 - 48 hours. At that stage, in 1991, thorascopic surgery was in its infancy, but I had the opportunity of a very helpful colleague (Schutte), an encouraging hospital management who assisted with the equipment, and patients who at all times were fully informed of the procedure and were aware that the accepted open procedure might be used if necessary. A trip visiting prominent units in the USA followed, which stimulated me to continue what I was doing.

The 1990s era was a time when we were taught:

- Keyhole surgery is bad surgery.
- Real surgeons make big cuts.
- You must be able to see what you are doing.

Surgery still says: You must see what you are doing. The better the vision, the better the surgery. Modern thorascopic equipment gives by far better visualisation than even the best loops and optics of the past. It also allows one to see around corners that you could not with open surgery. The instruments are constantly evolving, allowing the most complex procedures to be performed more safely than ever before.

## 1991: The beginning

The beginning was the learning stage. The equipment was one-chip technology and the screens were small and of inferior quality, but the experience was exhilarating. I was just one surgeon learning as I progressed. My trips to various units in the USA and Europe showed me that surgeons were in the learning phase everywhere. I visited the Royal Brompton Chest in London, Stuttgart Clinic in Germany, the Memorial Sloan Kettering Cancer Center, Philadelphia University, Methodist Dallas Medical Center and the Mayo Clinic in the USA, and since have visited many others, always learning something new. The experience convinced me that the future of surgery was minimally invasive. Bob Ginsberg at Sloan Kettering at the time said to me, ‘I do not believe in this new technology but will allow my junior staff to investigate it.’ Goldstraw at the Brompton had similar sentiments. This taught me the importance of a university investigating new procedures, but with, of course, checks and balances. These two men were real leaders who did not stop innovation but rather encouraged their juniors. History teaches us of the great scientists of the past who have been stifled because of the bureaucracy of the time. We are in the process of great change, in not only medicine but also many areas of the modern world. At our peril, we remain mired in the past. Continuing medical education is vital to remain current in our knowledge and in our practice of medicine.

During the 1990s, many thorascopic courses were held internationally, and I had the privilege of attending a number of these. At that time, the standard approach was to use three ports, working in a large triangle, with the camera pointing towards the lesion, and with two instruments, one in each hand. The camera pointed away from the surgeon. As more and more academic units adopted the use of thorascopy, the courses were gradually discontinued as residents and registrars were exposed to the techniques in their routine training.

My first cases were simple lung biopsies. These were followed by a mediastinal gland biopsy. Soon I had a number of recurrent spontaneous pneumothoraxes for which I performed a bullous resection and an apical pleurodesis. One of my very early patients was a businessman with enlarged mediastinal glands, who felt that he could not take time off work. He waited for me to return from one of my overseas trips, was admitted on the day of the procedure, had a biopsy at 11h00 h, was discharged at 18h00 and returned to work the following day. The surgery was performed on the Wednesday, and on the Saturday he played a round of golf and reported that he was two below his handicap. The difference between open surgery and minimally invasive thoracic surgery was like night and day. One patient had a lymphoma diagnosed without the trauma of a major operative procedure. An interesting case was the endoscopic division of an anomalous venous drainage in which the right subclavian artery arose distally from the aorta as the third branch winding behind the oesophagus. The vessel was ligated endoscopically, and attached in the neck to the common carotid through a small neck incision. This adult patient had presented with dysphagia that was totally relieved by the surgery.

At the time, unfortunately, there was significant resistance to thoracoscopy from a number of my colleagues, who felt that the dangers outweighed its advantages. The medical aid schemes complained about the cost. I was questioned about safety and about any advantages. It was pointed out that even a simple chest drain can cause a post-thoracotomy syndrome. My international trips, however, had convinced me of the benefits, and I continued the work. I soon had experience encompassing over 1 500 cases.

Thorascopic surgery is still in its very early stages in most SA academic units. The last few years, however, have seen the increasing introduction of video-assisted thoascopic surgery (VATS) techniques into our university departments. Very little thorascopic surgery, however, is being performed in sub-Saharan Africa outside SA. From my many visits and via personal communication, I can state that no thorascopic surgery is being performed in Nigeria, Kenya, Malawi, Tanzania, Zambia, Zimbabwe or Botswana.

## 2000: Maturing

By 2000, the terminology had changed. The term VATS had been introduced. The thoracoscope had become an adjunct to open thoracic surgery. It provides an eye and a light source inside the chest. As the surgeon becomes more skilled, the incisions become smaller and smaller. The massive aggressive incisions of the past have become historic techniques, and should only be found in museums and the history books of the great men and women who led our profession 70 - 100 years ago.

By 2000 I required thorascopic instruments to be available in my operating room whenever I performed a chest procedure other than a simple bronchoscopy or mediastinoscopy. A lobectomy become a minimally invasive procedure as I gradually decreased the size of my incisions. As the incisions become smaller, so did the degree of rib spreading, until eventually I was performing major resections with no rib spreading whatsoever. Most of the instruments for the resections were the usual thoracic instruments available in most general thoracic instrument trays. Very importantly, the patients were always draped for a thoracotomy.

At this stage, over 60% of all my cases that were previously performed through a thoracotomy were now VATS procedures.

VATS lobectomy had been proposed in the mid-1990s by McKenna and others.^[4]^ I began by using a video-assisted approach, gradually decreasing the size of my incision. One of the incisions needs to be big enough to remove the specimen, this being the so-called access incision. This incision is about 4 - 6 cm long, sometimes larger, depending on the size of the lesion to be removed. There is no point struggling with a small incision only to enlarge it to remove the specimen. My standard approach in the early days was a 3-port approach, with the larger anterior incision for the access port.

During 2015 I was invited to Prof. Gonzalez-Rivas’s unit in La Corona, Spain, where I witnessed the uniportal lobectomy.^[5]^ I subsequently spent 2 weeks in the Shanghai Pulmonary Hospital, observing not only the uniportal approach but highly skilled surgeons using various techniques, including the standard 3-port, a 2-port approach and of course the uniportal. I also observed a subxiphoid lobectomy and a subcostal incision. Thymectomy was observed through a right and a left thoracoscopy, as well as via the subxiphoid approach. These are new attempts to totally prevent any rib spreading. Even minimum spreading of the ribs, in removing the specimen, can cause a debilitating post-thoracotomy syndrome.

## 2008: Pectus surgery

By 2008 I was attending international cardiothoracic meetings. In 2008 was the World Society of Cardiovascular and Thoracic Surgeons congress on the island of Kos in Greece. At this meeting, Mustafa Yuksel from Istanbul presented his work on pectus excavatum. He was performing the Nuss procedure, which is a minimally invasive repair of the disorder. We became very friendly, and over dinner with our wives he invited me to visit his unit. Later that year that is exactly what I did. Over a week spent in the vibrant city of Istanbul in Turkey, I observed Prof. Yuksel operating, while learning how to do the operation myself. I was collecting cases, and in October 2008 I invited Prof. Yuksel to join me in Johannesburg, where we operated on four cases. Also present at that time was Prof. Pilegaard from Denmark, who had his own modification of the Nuss procedure. The following month I had a further four cases. To my delight, I discovered that Prof. Donald Nuss himself was visiting SA, and he kindly agreed to assist me with my cases. Prof. Nuss is a South African who qualified in Cape Town as a paediatric surgeon under Prof. Jannie Louw. Nuss spent time at the Mayo Clinic in the USA, following which he was offered a position in Norfolk, Virginia, USA. He began his research in minimally invasive repair of pectus excavatum in 1987.^[8]^ After his presentation in 1998, the repair of the disorder was revolutionised. Over the next few years, the VATS repair became the new standard replacing the Ravitch operation, which was the routine operation at the time.

I had met Prof. Ravitch as a registrar when he visited our unit in Cape Town in 1980. He was a truly amazing man, and the leading paediatric surgeon of his time. He gave a lecture on his procedure, but also mentioned his work on surgical staplers. He introduced surgical staplers to the West from the USSR (now Russia). His research on staplers was undertaken at the back of a friend’s dry-cleaning business, a Mr Hirsch, and this eventually became the company USSC (United States Surgical Corporation), which after many transitions is now Medtronic. A true innovator, I am sure he would have been at the forefront of research in the constantly changing field of surgery: When you think you know everything about your field of study, when there is nothing extra to learn, that is the time to retire.

My teachers have been most gracious in their sharing of knowledge. I will always be grateful to Yuksel, Pilegaard, Nuss and so many others who have freely passed on their surgical secrets, and in so doing affected the lives of literally thousands of patients.

During this period, I became a member of the Chest Wall Interest Group, which has now been reformatted as a society, the Chest Wall International Group (CWIG). As part of this group I have met many other truly innovative doctors. Prof. Horacio Abramson, after observing the Nuss procedure, said, ‘If the Nuss works for pectus excavatum, why not a reverse Nuss for the carinatum?’ And so, the Abramson procedure for the pectus carinatum was born.

External braces for pectus carinatum had been used for some time, but these had their own problems. Marcelo Martinez-Ferro introduced a custom-made adjustable brace with excellent results.

My own experience now includes 134 Nuss procedures for pectus excavatum, 8 Abramson procedures for pectus carinatum and 14 custom-made dynamic compression braces for pectus carinatum. My advice is the brace as the first option for carinatum, which has been shown to correct most defects, reserving surgery for cases where the patients are not willing to wear the device, some older patients, patients with a very high compression required to correct the problem and some complex cases where a Ravitch may be required.

## 2018: The future

We are now in the beginning of what I believe to be a new era in surgery, the era of robotic surgery. The discussion we are now in the middle of echoes almost identically what was said when thorascopic surgery was introduced in 1991:

- It is not safe! (Farjah^[6]^)
- It is expensive! (Casali^[7]^)
- There are no advantages!
- Complications are potentially disastrous!
- It is no better than thoroscopic surgery! (This being the new standard.)

The robot now available is the De Vinci. This is being constantly upgraded, the cost is rapidly coming down, many new companies are on the horizon and more and more articles are being published on the advantages of incorporating robotic technology in operations. Handheld robots will soon be available, allowing the surgeon to combine the technology of open, VATS and robotic techniques (personal observation, Israel).

## Discussion

Thoracoscopy can be divided into diagnostic procedures and therapeutic procedures. For pleural effusion, the sensitivity of thoracoscopy is 92% - 97%, and its specificity is 99%. Thorascopic lung biopsy has a similar sensitivity of over 90%.^[3]^ The diagnostic procedures can be performed under sedation and local anaesthetic, and in many cases the patients can be discharged on the day of the procedure. Table 1 gives my indications for diagnostic procedures.

**Table 1:** Diagnostic indications (as performed in the author’s practice)

Pleural biopsies
Lung biopsies
Mediastinal gland biopsies
Staging for lung cancer

The therapeutic procedures, more correctly in my view, should be referred to as VATS. If a thoracotomy is indicated, a VATS procedure is usually a consideration. Pleural adhesions are a relative contraindication. Flimsy adhesions can be broken down. Dense adhesions, as are often found in the African scenario, may be more difficult. In my learning phase, I used the thoracoscope as a light source, with the incision becoming smaller and smaller. My indications for VATS are given in Table 2.

**Table 2:** Therapeutic indications (as performed in the author’s practice)

Bullous ligation
Pleurodesis
Pleurectomy
Decortication
Lobectomy
Pneumonectomy
Mediastinal lymph node clearance
Oesophagectomy (chest part; abdominal mobilisation of stomach performed laparoscopically)
Ruptured diaphragm
Clotted haemothorax
Trauma
Thymectomy
Mediastinal masses
Posterior mediastinal masses
Sympathectomy
First rib resection
Pectus excavatum (Nuss procedure)
Pectus carinatum (Abrahams procedure)

Work is taking place on a subcostal as well as a subxiphoid approach, to further limit postoperative pain. At the Shanghai Pulmonary Hospital in China, I observed these procedures being performed with great skill.

Very importantly, the surgeon at all times must be ready to convert to an open thoracotomy. All my patients are draped and prepared for a thoracotomy.

Is it ethical to still be doing trials comparing thoracotomy to VATS? Many studies have been performed comparing anterolateral thoracotomy to VATS, and have shown a marked difference in the postoperative pain and quality of life after each type, confirming the superiority of VATS.^[10–13]^ The main reason not to perform VATS is probably the answer to the most important question that should be asked, which can be summarised as ‘unwilling to’ or ‘unable to’.^[9]^ Possible reasons for this are lack of experience, the cost of equipment and the culture and bias of the local unit.

There is a learning curve, especially for VATS lobectomy and thymectomies, but the simple procedures should be within the capabilities of all thoracic surgeons.^[14,15]^

## Conclusion

My prediction: We are in the middle of a revolution of new technology. New is not always better, but change is inevitable. As Africans, we have the capabilities to be equal to the best in the world. Our leading universities have the facilities to train the surgeons, we have the patients, and we most certainly have the young graduates who are more than capable of being the best in the world. I would encourage the newly qualified to spend time in leading international units. The time spent will broaden the mind, and is never wasted.

Thorascopic chest surgery is the modern standard. The teaching of thorascopic techniques should be standard in all our university departments. A newly qualified specialist thoracic graduate who is not trained to perform minimally invasive thoracic procedures is inadequately trained.

The next generation will slowly move to robotic surgery. We are now in the early phases, but the future is rapidly approaching. The other field that I believe has great potential is the field of 3D printing. This will involve prostheses as well as other equipment.

VATS allows the same operation to be performed safely, with less pain, shorter hospital stay and with outcomes that are in many cases better than those of open surgery.

The future is exciting. As Africans, we need to be, and can be, at the forefront of development.

## References

[1] Jacobaeus HC (1923). The cauterisation of adhesions in artificial pneumothorax treatment of pulmonary tuberculosis under thoracoscopic control.. Proc R Soc Med.

[2] Colt HG (1999). Thoracoscopy: Window to the pleural space.. Chest.

[3] Boutin C, Loddenkemper R, Astoul P (1993). Diagnostic and therapeutic thoracoscopy: Techniques and indications in pulmonary medicine.. Tuber Lung Dis.

[4] McKenna RJ, Houck W, Fuller CB (2006). Video-assisted thoracic surgery lobectomy: Experience with 1 100 cases.. Ann Thorac Surg.

[5] Gonzalez-Rivas D (2014). Single incision video-assisted thoracoscopic anatomic segmentectomy. Ann Cardiothoracic Surg.

[6] Farjah F, Wood DE, Mulligan MS (2009). Safety and efficacy of video-assisted versus conventional lung resection for lung cancer.. J Thorac Cardiovasc Surg.

[7] Casali G, Walker WS (2009). Video-assisted thoracic surgery lobectomy: Can we afford it?. Eur J Cardiothorac Surg.

[8] Nuss D, Kelly RE, Croitoru DP (1998). A 10-year review of minimally invasive technique for the correction of pectus excavatum.. J Pediatr Surg.

[9] Cheng-Che Tu, Po-Kuei Hsu (2016). The long-waited high level evidence in thoracic surgery.. Ann Transl Med.

[10] Bendixen M, Jørgensen OD, Kronborg C (2016). Postoperative pain and quality of life after lobectomy via video-assisted thoracoscopic surgery or anterolateral thoracotomy for early stage lung cancer: A randomised controlled trial.. Lancet Oncol.

[11] Yan TD, Black D, Bannon PG (2009). Systematic review and meta-analysis of randomised and nonrandomised trials on safety and efficacy of video-assisted thoracic surgery lobectomy for early-stage non-small-cell lung cancer.. J Clin Oncol.

[12] Cao C, Manganas C, Ang SC (2013). Video-assisted thoracic surgery versus open thoracotomy for non-small cell lung cancer: A meta-analysis of propensity scorematched patients.. Interact Cardiovasc Thorac Surg.

[13] Long H, Lin ZC, Lin YB (2007). [Quality of life after lobectomy for early stage nonsmall cell lung cancer-video-assisted thoracoscopic surgery versus minimal incision thoracotomy].. Ai Zheng.

[14] Petersen RH, Hansen HJ (2012). Learning curve associated with VATS lobectomy.. Ann Cardiothorac Surg.

[15] Zhao H, Bu L, Yang F (2010). Video-assisted thoracoscopic surgery lobectomy for lung cancer: The learning curve.. World J Surg.

[16] Boutin C, Cargnino P, Viallat PR (1980). Thoracoscopy in the early diagnosis of malignant pleural effusions.. Endoscopy.

